# Critical Assessment of Purification and Analytical Technologies for Enveloped Viral Vector and Vaccine Processing and Their Current Limitations in Resolving Co-Expressed Extracellular Vesicles

**DOI:** 10.3390/vaccines9080823

**Published:** 2021-07-26

**Authors:** Aline Do Minh, Amine A. Kamen

**Affiliations:** Department of Bioengineering, McGill University, Montreal, QC H3A 0E9, Canada; aline.dominh@mail.mcgill.ca

**Keywords:** extracellular vesicles, enveloped viruses, lentiviral vectors, viral vaccines, purification process, analytical technologies

## Abstract

Viral vectors and viral vaccines are invaluable tools in prevention and treatment of diseases. Many infectious diseases are controlled using vaccines designed from subunits or whole viral structures, whereas other genetic diseases and cancers are being treated by viruses used as vehicles for delivering genetic material in gene therapy or as therapeutic agents in virotherapy protocols. Viral vectors and vaccines are produced in different platforms, from traditional embryonated chicken eggs to more advanced cell cultures. All these expression systems, like most cells and cellular tissues, are known to spontaneously release extracellular vesicles (EVs). EVs share similar sizes, biophysical characteristics and even biogenesis pathways with enveloped viruses, which are currently used as key ingredients in a number of viral vectors and licensed vaccine products. Herein, we review distinctive features and similarities between EVs and enveloped viruses as we revisit the downstream processing steps and analytical technologies currently implemented to produce and document viral vector and vaccine products. Within a context of well-established viral vector and vaccine safety profiles, this review provides insights on the likely presence of EVs in the final formulation of enveloped virus products and discusses the potential to further resolve and document these components.

## 1. Introduction

Viral vectors and viral vaccines have been part of the medical landscape for decades, as approved products or under evaluation in numerous clinical trials. About 14% of vaccines approved by the FDA involve enveloped viruses [1], while out of the 15 gene therapy products approved worldwide in 2019, six of them use enveloped viruses [2], and 39% of gene therapy clinical trials are using enveloped viruses [3]. Enveloped viruses are encased in a lipid bilayer which, in most cases, fuses with the target host cell membrane to infect cells. These enveloped viruses are produced in various systems, including traditional embryonated chicken eggs or more advanced cell culture technologies such as MRC-5 cells, Vero cells and HEK293-derived cell lines. Table 1 summarizes vaccines and gene therapy products using whole enveloped viruses. The manufacturing of viral vector and viral vaccine products has always been paved with challenges related to the downstream processing. Purification process unit operations usually start with harvest and clarification, followed by intermediate purification steps, before polishing and formulation steps [4]. Although techniques have greatly improved over the years to generate purer high-quality products and reproducible processes while maintaining or decreasing the cost of goods, regulatory agencies are increasingly stringent regarding product identity and characterization of the end products and level of acceptable impurities as a way to ensure public safety and maintain public trust in this class of medicine.

Different biological systems are used to produce enveloped viruses. All of them, as with most cells and cellular tissues, secrete naturally extracellular vesicles (EVs). The interest towards those vesicles has recently increased as they may be used as therapeutic tools or biomarkers [10]. They are cell membrane-derived blebs that transport lipids, proteins and nucleic acids including DNA, mRNA, micro RNAs (miRNAs) and non-coding RNAs (ncRNAs). Their subpopulations are highly heterogenic in size and composition. EVs are extensively studied for their role in cell-to-cell communication and their ability to deliver their cargos from donor to recipient cells [11]. Exosomes and microvesicles are the most commonly cited EV subtypes [12]. Minimal information for studies of extracellular vesicles 2018 (MISEV 2018) [13] recommends classifying EVs by their physical characteristics (size or density), small EVs referring to particles smaller than 200 nm and medium/large EVs being larger than 200 nm. Other characteristics such as their biochemical composition or their subcellular origin have also been considered. The EV cargo composition depends on many factors, including the cell line from which they derive. However, the mechanism behind cargo sorting is still under careful investigation. Their budding pathways have also been analyzed. As EV subtypes do not have the same intracellular origin, their generation and release are not ruled by the same processes, even though they may share some mechanisms. Multivesicular bodies are formed from the fusion of endosomes, which derive from the invagination of the cell membrane, with molecular cargos sorted in the endoplasmic reticulum and processed in the Golgi complex. The lysosomal pathway leads to the degradation of the multivesicular bodies’ content upon fusion with lysosomes. In the secretory pathway, the content of the multivesicular bodies is released into the extracellular environment in the form of exosomes upon maturation and fusion to the plasma membrane. The key component for the exosome biogenesis within the endosomes is the endosomal sorting complex required for transport (ESCRT) [14]. The ESCRT molecular machinery includes four multi-protein complexes (ESCRT-0, -I, -II, -III) and associated accessory proteins Alix and VPS4.

The existence of an ESCRT-independent mechanism was also unraveled, potentially involving other partners such as heat shock proteins, cholesterol, tetraspanins, phosphatidic acids and ceramides [15]. The reason why some multivesicular bodies undergo the secretory pathway or the degradation pathway remains to be understood. The mechanism underlying the generation of microvesicles is also not well understood. It was demonstrated that ESCRT-I component Tsg101 was involved in protein sorting into microvesicles [16], confirming that mechanistic elements may be shared in exosome and microvesicle biogenesis.

Viruses, as per their nature, take over many functions of the cells they are infecting. Viral nucleic acids and viral proteins of many enveloped viruses have been incorporated into host EVs. For instance, HIV Nef protein can be incorporated into EVs [17], while HIV trans-activating response (TAR) element RNA was also detected in EVs [18]. It has been hypothesized that viruses hijack the host pathways for vesicle trafficking [19], and one cannot deny the similarities between the biogenesis of viruses and EVs due to the implication of common proteins such as the ESCRT machinery once again, SNARE, SNAP and the cargos resemblance [20]. 

**Figure 1:**
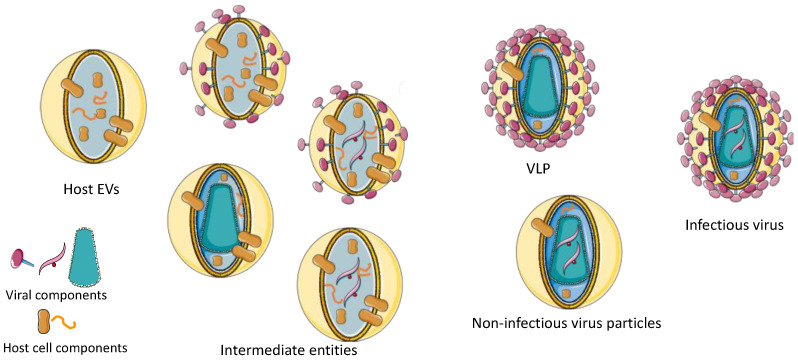
Expected EV, LV and intermediate entities during production of lentiviral vector (Figure created using Servier Medical Art by Servier). Viral components (left to right): envelope protein, viral genome, viral capside.

When it comes to viruses mixed with coproduced EVs, the distinction becomes even more challenging as EVs exist in a wide spectrum of populations, which is further broadened by virus production. EVs produced by cells that are also producing viruses likely contain viral proteins and parts of viral genetic material. Thus, it is reasonable to expect that, in the context of enveloped virus production, diverse vesicles are released. On one extreme, there are EVs that are entirely made of host cell components, while on the other extreme, there are infectious viruses. Ranging between these two entities, there are many intermediate structures, such as non-infectious particles that could be considered as incomplete viral particles or as EVs that have incorporated fragments of the viral genome and viral (glyco)proteins (Figure 1).

Few studies have been designed to compare viruses to coproduced EVs in cell culture produced systems using omics approaches [21,22]. Sviben et al. compare mumps and measles produced in Vero cells to the coproduced EVs, while Do Minh et al. compare lentiviral vectors to coproduced EVs in a HEK293-derived cell line. Do Minh et al. indeed identified subpopulations such as host EVs with or without the “viral genome”, non-functional LVs despite carrying the viral genome and fully functional LV particles. Other conclusions from both studies unsurprisingly reveal that EVs and the studied viruses share many features, including protein cargos, rendering specific markers difficult to establish. More studies on retroviruses [23] also associated CD63 with highly purified retroviral vectors, while the tetraspanin is often used as an exosome marker [24]. Some studies claimed the separation of HIV from coproduced EVs [25,26,27] using density gradients. Besides the similar density of HIV-1 and small EVs questioning the reliability of the method, the separation process used is also far from being ideal for large scale manufacturing. Table 2 describes the size range of EVs and how they compare to other particles such as viruses.

Similar downstream process unit operations are used in both fields. The isolation of enveloped viruses and EVs was for instance traditionally achieved using ultracentrifugation. More advanced techniques including chromatography and filtration are being increasingly developed. However, it is likely that the presence of EVs is largely unassessed and undocumented so far in the manufacturing of enveloped viruses, since the composition of EVs greatly resembles that of the targeted viral product.

In this review, we go through distinctive features and similarities between EVs and enveloped viruses as we describe the downstream processes and analytical methods currently used in the production of viral vectors and vaccines. Large scale technologies used in the field of viral vectors and vaccines for the purification of enveloped viruses are the main focus of this review. To assess the process reproducibility and robustness, analytical tools used for characterizing the critical quality attributes of the final viral products are also reviewed.

## 2. Viral Purification Processes

**Figure 2:**
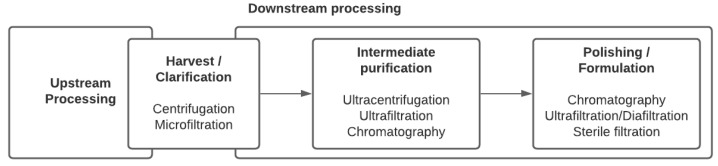
General sequence of viral vector and viral vaccine production bioprocesses.

No unique stream exists in the downstream processing of viral vectors and vaccines. Indeed, not only does each virus have its own properties and behavior, but the treatment that viruses can undergo also depends on the nature of the final product: Should the virus be inactivated or live-attenuated retaining infectivity properties, does the particle structure have to be maintained for immunogenicity, or should the virus, that might be defective, retain the properties to effectively transduce cells and express the targeted transgene, as is the case for viral vectors used in gene therapy or vaccination? Traditional techniques for purifying EVs and viruses involved ultracentrifugation and filtration and are still being used extensively. However, more advanced chromatographic steps and scalable technologies are being implemented. Common steps and process unit operations are presented in a generic sequence summarizing the purification strategy (Figure 2). In the case of enveloped viruses, lysis of the cells is not required as the viruses bud out of the cell membranes. Therefore, the first clarification step aims at removing cells and cell debris. Centrifugation and filtration are the most commonly used techniques at this stage. In general, one or multiple purification steps follow in order to concentrate the virus and remove host cell proteins (HCPs) and host cell DNA. They might include tangential flow filtration and chromatographic unit operations. Buffer-exchange steps and nuclease treatment steps are often required at different downstream process stages. Below, we describe the general sequences of enveloped virus purification streams using (ultra-) centrifugation and various filtration and chromatography techniques.

### 2.1. Harvest and Clarification

In both cell culture or egg production systems, the transition step between upstream and downstream processing is known as the harvest. As stated, in the case of enveloped viruses or EVs, since particles directly bud out of the cells, there is no need to use detergents to lyse the cells. Therefore, downstream processing starts directly with removing cells and large debris. It is often completed in two steps, combining centrifugation and filtration.

#### 2.1.1. Centrifugation

Centrifugation is a common way to remove cells and large cellular debris by pelleting them. It is still used broadly despite the high cost and difficulty to scale up as it offers good recovery [28]. Based on their lower density, viruses and EVs are therefore both recovered in the supernatant during this step.

#### 2.1.2. Microfiltration

Microfiltration is referred to using filters with membrane cut-offs usually between 0.1 and 10 µm. Different filtration techniques are used with such filters, the main ones being normal-flow filtration (NFF) and tangential flow filtration (TFF). TFF, according to its name, differs from NFF in the flow directionality. Both have been extensively and very efficiently used in the separation and purification of biotherapeutics.

Different types of filters can be used in NFF: dead-end filters and depth filters. Dead-end filters have defined pore sizes, and excluded particles are retained only at the surface, whereas depth filters are made of porous material, which can retain particles of different sizes across the membrane’s thickness [29], rendering membrane fouling less problematic. Depth filters can also be positively charged to effectively capture host cell DNA and HCPs. Both types of filters have the advantage of being easy to scale up and cost-effectiveness. A scalable process using a dual 0.45–0.2 µm filter has been proposed to clarify retroviral vectors produced in HEK293-derived cells [30]. NFF has also been used effectively for decades in the clarification process of influenza virus produced in embryonated chicken eggs [31].

In TFF, biological fluids recirculate in parallel to the membrane surface, preventing cake formation. Particles smaller than the pore size flow through the membrane in the permeate, while larger particles are retained by the membrane and are recovered in the retentate. TFF is also a highly scalable method and has been successfully implemented in the manufacturing process of smallpox and monkeypox vaccine JYNNEOS [32].

Given that these filters separate particles mainly based on their size and given the overlapping size range of viruses and EVs, both types of particles remain in the filtrate or permeate during this step.

### 2.2. Concentration and Intermediate Purification

#### 2.2.1. Traditional Ultracentrifugation

Traditional techniques for virus separations were based on their physical characteristics such as their size and density. Ultracentrifugation is a well-established technique that has been used for decades to pellet low-density particles. It can be used in one step, stepwise (differential (ultra-) centrifugation), with continuous density gradients or discontinuous density gradients called cushions with media such as cesium chloride, iodixanol or sucrose.

In the field of gene therapy, lentiviral vectors have been concentrated and partially purified using ultracentrifugation [33]. Iodixanol gradients [34] or sucrose cushions [35] have been used for purifying lentivirus preparations. Ultracentrifugation steps using sucrose gradients have also been used to purify retrovirus [36].

In the field of vaccines, ultracentrifugation and zonal-rate separation on sucrose cushions has been used widely for the purification of influenza virus. The FluMist vaccine, for example, employs ultracentrifugation in the production process. Japanese Encephalitis virus for the preparation of Ixiaro vaccine is purified using sucrose density gradients [37].

In the field of EVs, differential ultracentrifugation was for a while the gold standard to isolate EVs [38], with sequential steps of increased centrifugal force. It is no longer the method of choice, however, as it is cumbersome, induces higher variability than other techniques and has been shown to damage and aggregate EVs [39]. Ultracentrifugation with sucrose or iodixanol gradients is another popular approach to isolating EVs [40].

Ultracentrifugation using continuous density gradients is limited by the volume that can be processed (usually less than 50 mL); it is therefore mostly used for pre-clinical material or small-scale research samples. Continuous-flow centrifugation overcomes this volume limitation and is still being used at a large scale in vaccine manufacturing, especially in the case of influenza vaccine and Japanese encephalitis vaccine. However, it does not translate well for lentiviral vectors, which are subject to a loss of infectivity by ultracentrifugation with or without sucrose gradients [41]. Continuous-flow centrifugation equipment is also high-maintenance, costly and voluminous. Moreover, due to the overlapping density of EVs and viruses (Table 2), effective separation cannot be achieved. Ultracentrifugation is therefore not a suitable method to separate enveloped viruses from EVs, and viral manufacturing processes that rely on ultracentrifugation in downstream processing should expect retention of EVs in the bulk product if an additional step segregating the two entities is not considered.

#### 2.2.2. Ultrafiltration Tangential Flow Filtration

Ultrafiltration (UF) is another membrane separation technique with tighter pore sizes than in microfiltration, usually ranging from 1 to 100 nm. It is most commonly used in TFF mode to concentrate the products of interest, and combined with diafiltration (DF), it allows buffer exchange. This well-controlled and scalable technology induces very low shear stress, which makes it very popular in various biomanufacturing processes. UF/DF is widely used in the field of influenza virus production [42] using different membrane molecular weight cut-offs (MWCO), from 100 to 750 kDa. UF/DF is also a method of choice in the purification of retroviral and lentiviral vectors using 100 to 300 kDa membranes [43,44]. UF/DF has also been employed in the field of EVs, especially for its scalability advantage [22,45].

In UF/DF TFF, most viruses and EVs are larger than the molecular weight cut-off of the membrane. As they have very similar size ranges, as in any filtration technique, they are both recovered in the same phase, here in the retentate, while smaller particles pass through the permeate. Therefore, TFF cannot be used for separating EVs from enveloped viruses.

#### 2.2.3. Chromatography

Chromatography is a commonly used process unit in the downstream processing of viral vectors and viral vaccines. Its role is to capture the particle of interest (bind-elute mode) or impurities (flow-through mode). If the virus is bound to the chromatographic material, it is then eluted, allowing its purification and concentration. Separation by chromatography is based on the physicochemical interactions of the particles of interest with the solid phase in opposition to the contaminants or impurities.

Different supports exist for the solid phase, also called stationary phase.

The most traditional one is resin-based chromatography, using packed-bed columns of microbeads with specific chemical properties. Packed-bed chromatography is, however, mainly used in small molecule purification such as antibodies, as the larger size of viruses affects their diffusion into the pores of the adsorbent resin thus reducing the dynamic binding capacity.

Alternative chromatographic supports are convective media such as membrane adsorbers and monoliths through which processing time, capacity and recovery are improved for viral processes rendering them more cost effective. Membrane adsorbers are a combination of liquid chromatography and membrane filtration [46]. They offer reduced diffusion times compared to packed-bed chromatography, with high flow rate operation capabilities and low pressure drops. The low dead volume of the system also yields reduced buffer consumption. Although reusability is always an option, another advantage of membrane adsorbers resides in their suggested single-use format, removing the need for lengthy validated clean-in-place and regeneration procedures and eliminating the risk of cross-contamination. Membrane adsorbers have been successfully used for scalable processes of lentiviral and retroviral vectors with high titers [47].

The disposability advantage also goes for monoliths. Monoliths, also known as convective interaction media (CIM) are made of porous materials organized in a single block with highly cross-linked macropores with a diameter range of 10 to 4000 nm [48]. Similar to membrane chromatography, mass transport is essentially convective, allowing high flow rates and low pressure drops. Most chromatographic monoliths are made of polymethacrylate material and are operated at a large scale with radial flow devices. Monoliths have shown great performance in the purification of influenza virus and lentiviral vectors as compared to other chromatographic means [49]. Rubella virus is another example of an enveloped viral vaccine and has been efficiently concentrated and purified using a monolith with almost 100% recovery and maintained infectivity [50].

Chromatographic materials can also be characterized by surface chemistry. Ion-exchange, hydrophobic interaction, affinity, size-exclusion and mixed-mode are the main types.

Ion-exchange chromatography (IEX) is the most commonly used technique and is based on difference in charge between the viral envelope and the stationary phase. It is mostly operated in bind-elute mode. IEX usually offers high resolution especially when using elution gradients to fractionate closely related biomolecules. Depending on the particle of interest’s net charge, either anion or cation exchange is employed. Most viruses are negatively charged at physiological pH due to their isoelectric point (pI) being below 7.4. Interestingly, egg-derived influenza virus has been purified by both anion-exchange (AIEX) and cation-exchange (CIEX) chromatography, although AIEX was more favorable [42]. Lentiviral vectors, as well as retroviral vectors have been purified at a large scale using AEX, yielding 22% to over 60% of recovery of infectious particles [33,43].

The use of hydrophobic interaction chromatography (HIC) is scarcer. It is mainly known for the purification of vaccinia virus [51]. The reason behind the low popularity of the method is due to the high salt concentration used for desorption, which can be detrimental to the virus integrity and functionality, especially in the case of viral vectors used in gene therapy.

Affinity chromatography (AC) separation is based on specific interactions between the particles of interest and the stationary phase and is used in bind-elute mode. It has attracted interest in recent years. The advantage of AC is the high specificity of the interaction, yielding highly pure product in one step. Mechanisms of affinity include specific antigen–antibody interactions, which, when employed for the purification of measles virus [52], outperformed ultracentrifugation. Mumps virus purification using AC with a monolithic column coupled with polyclonal antibodies is another example [53]. Lectin affinity chromatography uses lectin ligands binding to specific carbohydrates via carbohydrate recognition domains. It was used in the purification of influenza A virus [54] and in the purification of HSV-1 [55]. Immobilized metal affinity chromatography (IMAC) is based on metal ion affinity such as zinc, cobalt, nickel or a combination of copper, cobalt and nickel and is used for the purification of influenza virus [56], HSV-1 [57], retroviral vectors [58] and lentiviral vectors [59], respectively. An additional example of AC mechanism is based on heparin affinity and has been very popular for the purification of many enveloped viruses, including HSV-1, vaccinia Ankara virus and retroviral and lentiviral vectors [60]. Despite the great performance of AC, it is not often implemented at a large scale due to the high cost of ligand design and immobilization.

Mixed-mode chromatography (MMC) is based on the combination of various multimodal binding mechanisms, such as ligands combining ionic interaction, hydrophobic interaction and hydrogen bonding. Hydroxyapatite, a complex crystalline compound, which resin binds at the same time negatively charged phosphate groups and positively charged functional groups, is a good example of MMC. It has shown recovery of up to 46% in the purification of retroviral vectors [61].

In the field of EVs, the use of AIEX has also been reported to efficiently isolate EVs from HEK293T cell cultures [62]. No studies attempting to separate viruses from coproduced EVs have been reported thus far. Despite the sizeable challenge, as charges between intermediate entities can exhibit slight differences, the possibility thus remains that this technique could separate EVs from viruses, as a recent study in another context showed the feasibility of separating full and empty adeno-associated virus (AAV) capsids [63].

When approaching AC techniques in the field of EVs, immunoaffinity appears appealing and has been widely employed in the isolation of EVs from cell culture or body fluids [64]. Tetraspanin proteins found at the surface of EVs are often reported as target molecules. However, specificity has not been demonstrated for efficient separation of EVs from viruses. Indeed, tetraspanins were also associated with viruses, such as CD63 with retroviral vectors and CD81 with lentiviral vectors [22,23]. Distinctive markers have yet to be accurately identified, and they may differ depending on the expression system and the produced enveloped virus. As documented in previous studies, EVs coproduced with enveloped viruses carry similar membrane proteins, and more extensive studies would be needed to identify and validate any specific markers that would enable separation of these two entities.

### 2.3. Polishing

Polishing is one of the last steps in bioprocessing, allowing the removal of remaining impurities, and can be completed after the final formulation of the product. This step is critical as it should ensure the purity, quality and potency of the final product according to stringent regulatory requirements.

Size exclusion chromatography (SEC) is the most commonly used chromatographic technique, based on the molecular size difference between the particle of interest and the impurities. SEC is used for example in the late-stage purification of lentiviral vectors [65]. Although still broadly used, SEC induces dilution of the final product and has usually low capacity.

Another MMC example used for polishing is the combination of size exclusion and binding properties of the Capto™ Core 700 and 400 resins. These are used in flow-through mode as the particles of interest are recovered in the flow-through, while impurities bind to the high-capacity column. It was originally designed for the removal of ovalbumin in the purification of influenza virus produced in eggs [66] but has since been applied to other viruses such as lentiviral vectors [65].

Polishing can also be achieved by UF/DF, which is covered in Section 2.2.2.

SEC and more recently Capto™ Core have been successfully implemented in the isolation processes of EVs [45,64,67].

Both are well-controlled technologies and scalable; however, since their separation principle is based on size, they cannot efficiently separate EVs from viruses due to their size similarities.

## 3. Analytical Tools in Virus Production

Process analysis technology deployment is critical for effective bioprocess development. Importantly, the final product destined for vaccine and gene therapy applications needs to be adequately characterized to ensure that it meets the claimed identity, purity, safety, quantity and potency. Analytical tools should have the ability to characterize the final product but also to monitor the performance of the bioprocess, showing it is robust and well-controlled. The need for advanced analytical technologies has been emphasized in recent years as the critical quality attributes of biologics have been refined. Measurements made throughout the process have to be reliable, accurate and reproducible. A good overview of assays used in virus-based therapeutics has been recently published [4] (Table 3). Identity of viruses can be determined by sequencing the genome DNA, identifying the viral proteins by Western blot or mass spectrometry, or confirming the isoelectric point if known. Purity assessment is usually related to impurity quantification, such as HCPs or HC-DNA, the quantities of which are strictly regulated in viral vaccines. Safety of the final product measures the level of microbial contaminants using bioburden and sterility tests, endotoxins and mycoplasma. Quantity is an attribute that is especially monitored throughout the process in intermediate products as well as in the end product, allowing the evaluation of the efficiency of each process unit. Total viral particles and vector genome particles are measured, as well as infectious particles in the case of viruses that need to retain infectivity, whereas transduction efficiency and expression of transgene are measured with defective viral particles. More generally, functional activity determining potency of the product can be assessed with cell-based or in vivo assays. In the following section, the most relevant analytical techniques used in the field of viral vectors and viral vaccine manufacturing are summarized. Some of these techniques can be applied to EV characterization, and their potential to segregate between EVs and viral entities is discussed.

### 3.1. Identity and Purity

Sodium dodecyl sulfate polyacrylamide gel electrophoresis (SDS-PAGE) is a well-established technique to determine purity of a bioproduct, using different staining strategies to reveal proteins present in the viral preparation. It can be combined with Western blotting to identify specific viral proteins. MS also allows the identification of viral proteins and residual HCPs. ELISA tests are available for both HCP and specific viral antigen quantification.

Host cell components including HCPs can also be associated with enveloped viruses as shown by Segura et al. [23]. Which HCPs are attributed to incorporation by the viruses or by the presence of EVs has yet to be resolved. Similarly, viral proteins could also modify host cell EVs. HCPs and viral protein-based assays can therefore be biased by the presence of EVs, viruses and intermediate entities.

Electron microscopy techniques are useful to visualize virus structural integrity. Full and empty particles can be distinguished by negative staining. Viruses with distinctive shapes can easily be distinguished from EVs. Recent approaches aimed at exploiting data from transmission electron microscopy (TEM) to achieve quantitative analysis [68]. The principles are based on shape, rendering the distinction between viruses and EVs difficult in the case of close shaped particles such as lentiviral and retroviral vectors. Moreover, the technique would need extensive optimization and standardization as it is subjective due to operator handling, reducing its reproducibility [69].

### 3.2. Quantity and Potency

Quantification of viral particles has always been a challenge. Orthogonal methods relying on different technologies and measuring different aspects of the virus complement each other to deliver more accurate measurements. Depending on the virus, different approaches are used.

Quantification of particles based on the presence of the viral genome can be achieved using polymerase chain reaction (PCR) methods, including real-time PCR (qPCR) and more recently droplet digital PCR (ddPCR), which eliminates the need for a standard curve. ddPCR has for instance been developed for the quantification of the influenza virus [70], lentiviral vectors [71] and VSV-based vaccine [72]. However, PCR techniques are based on specific primers, which have to be designed in such a way that they are specific to the measured particles. In Do Minh et al. [22], primers used in ddPCR targeted the woodchuck hepatitis virus post-transcriptional regulatory element (WPRE), which is commonly used in viral vector design to enhance transgene expression, the presence of WPRE thus indicating the presence of the viral genome. The cell line used in the study expressed the green fluorescence protein (GFP) transgene constitutively. In the absence of lentiviral vector production, ddPCR still yielded a titer when measuring isolated EVs, revealing that host EVs did incorporate sequences of the viral genome. The development of PCR-based methods needs therefore to be designed in such a way that viral specific elements are measured in order to distinguish coproduced EVs.

Physical quantification of viruses enables fast enumeration of total particles. Methods include nanoparticle tracking analysis (NTA), tunable resistive pulse sensing (TRPS) and multi-angle laser light scattering (MALLS) coupled with asymmetrical field flow fractionation (FFF-MALLS). NTA, based on the Brownian motion of particles in suspension, and TRPS, measuring transient change in electrical resistance as particles pass through the nanopore proportionally to their size [73], can also estimate particle size distribution. In FFF-MALLS, particles are eluted in order of size and simultaneously detected by light scattered from different angles. All techniques have been used in the field of viral vectors [33], vaccines [74] and EVs [75]. Although their detection methods differ, they are all based on particle size, in their lower limit of detection range for NTA and TRPS, rendering the distinction between viruses and coproduced EVs not possible.

Chromatography-based techniques using high-performance liquid chromatography (HPLC) present several advantages in terms of speed, accuracy and reproducibility for measuring total particles [49]. Different chemistries on different chromatographic supports can be used, such as AIEX and SEC on a favored monolith. Intact virus particles can be separated from other cellular impurities or incomplete virus particles. This approach creates a quick picture of a process step and an impurity profile of the intermediate product. One drawback of HPLC when intended for accurate virus quantification is that it requires highly pure and fully characterized virus material to develop the method and the reference material stock. HPLC has been developed for the quantification of the influenza virus [76], retroviral vectors [77] and lentiviral vectors [78]. Although the last of these studies did acknowledge the presence of EVs in lentiviral preparations, and the method was optimized in order to minimize their effect on the quantification of lentiviral vectors, the actual proportion of EVs in the final product could not be estimated as the analysis of a sample with no virus fell below the linear range of the method. The use of HPLC to quantify EVs and viruses cannot be excluded, but extensive optimization is expected, as shown in the field of AAV where full and empty capsid could be identified by AIEX-HPLC [79].

New technologies are being developed, including the ViroCyt virus counter. The proprietary technology uses a double fluorescence staining strategy: staining viral genomes (and nucleic acids in general) and viral capsids proteins, thereby allowing specific detection of the particles. Its performance showed a good correlation compared to other quantification methods in the quantification of filovirus [80] and vaccinia virus [81]. The equipment design is based on flow cytometry principles, and the staining strategy can be applied to more generic flow virometry. Compared to flow cytometry, the term flow virometry refers to the nanoscale operation of the equipment [82]. In flow cytometry for cells, the threshold is normally set to light scatter, with the light scatter triggering the detection. However, in the case of 100 nm particles, forward-scattered light (FSC) would not differentiate these particles from noise. A difference can be seen with side-scattered light (SSC); however, in order to reduce noise, increasing the FSC threshold would lead to loss of the signal of the targeted particles. Using fluorescence-triggered detection overcomes that issue. Flow virometry has been used in the last decade to quantify different viruses such as HSV-1 [83], vaccinia virus [84] and retrovirus [85]. It is also a method of choice for the quantification of EVs [86]. The staining strategies play a crucial role in flow virometry as they allow the detection and quantification of subpopulations. Although the use of dyes or stains requires careful optimization, the technology could allow the distinction between EVs, viruses and intermediate populations to some extent.

Many other assays are used in the field of viral vaccines and viral vectors, including more virus specific assays such as the hemagglutination assay, single radial immunodiffusion (SRID) used in influenza vaccine production and cell-based assays used to determine infectivity or functionality of the virus, such as the tissue culture infective dose assay (TCID50) or the gene transfer assay (GTA). All these methods are of utmost importance in bioprocesses but are not discussed as they cannot contribute to estimating the proportion of copurified EVs in the final product.

## 4. Conclusions

The field of viral vectors and viral vaccines is expanding, motivating the development of advanced bioprocesses for their large-scale manufacturing. The translation to the clinic of these complex biomolecular structures for treatment and prevention of diseases is challenged by the new findings in the emerging field of EVs that share many features with enveloped viruses from their physical characteristics to their biogenesis.

Many process units used for the purification of viruses have been adapted to EV isolation and purification. This is an early indicator that both particles are likely to behave similarly in a cell culture environment, and therefore it is expected that EVs copurify in the case of enveloped virus production. This review sheds light on the current unlikelihood of EVs to be effectively separated from cell culture-produced enveloped viruses by current large-scale bioprocesses, according to their similar characteristics in terms of size, density, charge and composition. The proportion of EVs in viral preparation also remains difficult to estimate as only a few methods show premises of capability to quantify both entities accurately using the same assay. The challenge is enhanced by the heterogeneous nature of both viral particles and EVs, which constitute more of a spectrum of populations rather than two distinct entities. Intermediate populations are therefore also more difficult to estimate, and their variations are manifold. The fact that EVs cannot be currently fully separated from viruses does not mean that they pose safety concerns as, on the contrary, they could serve as natural adjuvants in vaccine formulation. However, as per regulatory requirements, any component of the bulk medicinal product has to be carefully characterized, and EVs in enveloped virus preparations should not be an exception. Better analytical tools are therefore needed to gain expert knowledge on the actual proportion of EVs and intermediate entities in viral products.

## Figures and Tables

**Table 1:** Examples of approved vaccines and gene therapy products using whole enveloped viruses. VSV: vesicular stomatitis virus, JEV: Japanese encephalitis virus, VZV: varicella-zoster virus, YFV: yellow fever virus, HSV-1: oncolytic herpes simplex virus-1.

	Virus	Trade Name	Manufacturer	Production System	Target Disease/Indication	Reference
**Viral vaccine**	VSV	ERVEBO	Merck Sharp & Dohme (MSD)	Vero cells	Ebola	[1]
	Influenza virus	FluMist	Medimmune	Specific pathogen-free (SPF) eggs	Influenza	[1]
	JEV	Ixiaro	Valneva Austria GmbH	Vero cells	Japanese encephalitis	[1]
	Measles virus	M-M-R II ProQuad	MSD	Chick embryo cell culture	Measles	[1]
	Mumps virus	M-M-R II ProQuad	MSD	Chick embryo cell culture	Mumps	[1]
	Rubella virus	M-M-R II	MSD	WI-38 human diploid lung fibroblasts MRC-5 cells	Rubella	
		ProQuad				[1,5]
		Rudivax	Sanofi Pasteur MSD			
	VZV	ProQuad	MSD	MRC-5 cells	Varicella	[1]
		ZOSTAVAX				
		VARIVAX		WI-38 human diploid lung fibroblasts		
	Vaccinia virus	JYNNEOS	Bavarian Nordic A/S Emergent Product Development Gaithersburg, Inc.	Primary chicken embryo fibroblast cells Vero cells	Smallpox	[1]

		ACAM2000				
	YFV	YF-Vax	Sanofi Pasteur, Inc	Avian leukosis virus-free chicken embryos	Yellow fever	[1]
**Gene therapy**	Lentivirus	KYMRIAH	Novartis	HEK293-derived cells	Precursor B-cell lymphoblastic leukemia-lymphomaBeta-thalassemia	[6]
		Zynteglo	Bluebird bio	HEK293-derived cells		[7]
	Retrovirus	Strimvelis		HEK293-derived cells	Severe combined immunodeficiency	[8]
		Zalmoxis		HEK293-derived cells	Adjunctive treatment in haploidentical HSC transplantation	[9]
		YESCARTA		HEK293-derived cells	Lymphoma	[6]
	HSV-1	IMLYGIC		HEK293-derived cells	Melanoma	[6]

**Table 2:** Physical characteristics of extracellular vesicles and some enveloped viruses. VSV: vesicular stomatitis virus, HSV-1: oncolytic herpes simplex virus-1.

	Particle	Size Range	Density
**EVs**	Exosome	30–150 nm	1.13–1.21 g·ml^−1^
	Microvesicle	100–1000 nm	1.03–1.08 g·ml^−1^
	Apoptotic body	50–5000 nm	1.16–1.28 g·ml^−1^
**Enveloped viruses**	VSV	70–170 nm	1.19–1.20 g·ml^−1^
	Influenza A virus	80–120 nm	1.2 g·ml^−1^
	Lentivirus	80–100 nm	1.16–1.18 g·ml^−1^
	γ-retrovirus	80–120 nm	1.15–1.17 g·ml^−1^
	HSV-1	155–240 nm	1.27 g·ml^−1^

**Table 3:** Analytical assays most commonly used in-process and with end product in enveloped viral vector and vaccine manufacturing (adapted from Moleirinho et al. [4]).

Critical Quality Attribute	Assay (Parameter)
Identity	PCR-based assay (genomic DNA) Western blot (viral protein) Sequencing (genomic DNA) Mass spectrometry (viral protein) Isoelectric focusing (isoelectric point)
Purity	Electron microscopy (viral structure) ELISA (residual HCPs) Mass spectrometry (residual HCPs) PCR-based assay (residual HC-DNA)
Safety	Bioburden (microbial contaminants) Sterility test (microbial contaminants) Endotoxin assay (endotoxin) Mycoplasma testing (mycoplasma)
Quantity	PCR-based assay (vector genome particles) Plaque assay (infectious particles) TCID50 (infectious particles) ELISA (total vector particles) NTA (total vector particles) TRPS (total vector particles) FFF-MALS (total vector particles) Flow virometry (total vector particles)
Potency	Cell-based assay (functional activity) In vivo assay (functional activity)
